# Boda Bodas and Road Traffic Injuries in Uganda: An Overview of Traffic Safety Trends from 2009 to 2017

**DOI:** 10.3390/ijerph17062110

**Published:** 2020-03-22

**Authors:** Silvia D. Vaca, Austin Y. Feng, Seul Ku, Michael C. Jin, Bina W. Kakusa, Allen L. Ho, Michael Zhang, Anthony Fuller, Michael M. Haglund, Gerald Grant

**Affiliations:** 1 Department of Neurosurgery, Stanford University School of Medicine, Stanford, CA 94305, USA; svaca@stanford.edu (S.D.V.); fenga@stanford.edu (A.Y.F.); skku@stanford.edu (S.K.); mjin2@stanford.edu (M.C.J.); bkakusa@stanford.edu (B.W.K.); aho5@stanford.edu (A.L.H.); zhangm@stanford.edu (M.Z.); 2 Stanford Center for Global Health Innovation, Palo Alto, CA 94305, USA; 3 Division of Global Neurosurgery and Neurology, Department of Neurosurgery, Duke University, Durham, NC 27710, USA; anthony.fuller@duke.edu (A.F.); michael.haglund@duke.edu (M.M.H.)

**Keywords:** Uganda, road traffic injury, motorcycle, Africa, trauma, road safety, traumatic brain injury

## Abstract

Introduction: Road traffic injuries (RTIs) are an important contributor to the morbidity and mortality of developing countries. In Uganda, motorcycle taxis, known as boda bodas, are responsible for a growing proportion of RTIs. This study seeks to evaluate and comment on traffic safety trends from the past decade. Methods: Traffic reports from the Ugandan police force (2009 to 2017) were analyzed for RTI characteristics. Furthermore, one month of casualty ward data in 2015 and 2018 was collected from the Mulago National Referral Hospital and reviewed for casualty demographics and trauma type. Results: RTI motorcycle contribution rose steadily from 2009 to 2017 (24.5% to 33.9%). While the total number of crashes dropped from 22,461 to 13,244 between 2010 and 2017, the proportion of fatal RTIs increased from 14.7% to 22.2%. In the casualty ward, RTIs accounted for a greater proportion of patients and traumas in 2018 compared to 2015 (10%/41% and 36%/64%, respectively). Conclusions: Although RTIs have seen a gross reduction in Uganda, they have become more deadly, with greater motorcycle involvement. Hospital data demonstrate a rising need for trauma and neurosurgical care to manage greater RTI patient burden. Combining RTI prevention and care pathway improvements may mitigate current RTI trends.

## 1. Introduction

Road traffic injuries (RTIs) are a serious source of morbidity and mortality in the world. According to the WHO, motor vehicle crashes in 2016 claimed an estimated 1.35 million lives [1]. These deaths now outnumber those caused by traditional communicable diseases like tuberculosis or HIV/AIDS [1]. RTIs disproportionately affect low- and middle-income countries (LMICs), where more than 90% of road traffic deaths occur despite only 60% of global vehicle possession. The economic burden of these injuries can cost up to 10% of the yearly gross domestic product [1,2]. As the leading cause of death of youth (ages 5–29), RTIs affect a productive segment of the labor force and have lasting impacts on the country’s future [3]. With the increased use of motor vehicles that follows economic growth, it is not surprising that RTIs are projected to be the third leading cause of disability-adjusted life years (DALYs) lost by 2020 [4]. Thus, it is imperative to better understand RTIs in LMICs in order to focus preventative efforts to reduce their incidence.

In Uganda, motorcycle taxis—known as boda bodas—are a mainstay of transportation; in 2009, an estimated 100,000 Ugandans operated boda bodas [5]. Studies from Uganda and neighboring Tanzania show boda bodas are responsible for up to 58.8% of RTIs, leading to mortality and long-term morbidity [5,6,7,8,9]. Additionally, boda boda RTIs are costly from a societal perspective; more than half of the surgical budget for Mulago National Referral Hospital (MNRH), Uganda’s largest public hospital, has been dedicated to boda boda injuries [10]. 

Numerous measures may help reduce RTIs, such as road quality improvements, speed bumps, posted speed limits [11]. However, despite their relative cost-effectiveness, difficulties with funding and enforcement currently make infrastructural improvements less appealing [12]. An alternative is improving personal safety, particularly helmet use. For motorcyclists, meta-analyses have shown helmets to be effective in reducing death by 42% and head injury by 69% in RTIs [13]. Unfortunately, there is poor helmet usage among boda boda drivers. Various studies of Ugandan emergency departments have shown only 18.6% to 22% of motorcyclists wore helmets [5,14]. Given the serious cost of RTIs and the relatively low cost of helmets, this discrepancy reflects an important area that public health should address.

As Uganda continues to grow, the number of boda bodas on the road will undoubtedly increase. This reality highlights the necessity and urgency of addressing the major road safety issues revolving around boda boda drivers. This study seeks to provide an overview of Ugandan traffic safety trends in the past decade, with a special focus on boda bodas and recommendations for decreasing RTI morbidity and mortality. 

## 2. Materials and Methods

### 2.1. Ethical Approval

Ethical approval was provided by the Stanford University Institutional Review Board (IRB) in the United States and the Makerere University IRB in Uganda (protocols 36749 and 2016077, respectively).

### 2.2. Police Reports

Annual traffic reports from the Ugandan police force from 2010 to 2017 were analyzed for trends in RTIs [15,16,17,18,19,20,21,22]. Extracted data include casualties by age and gender, crash distribution with respect to time, regional distribution of RTIs, vehicular involvement, RTI causes, and RTI severity. As defined by the traffic reports, serious RTIs are defined as causing serious (i.e., permanent disfiguring or disabling) or minor (i.e., bodily injury not severe enough to require roadside assistance) injuries to at least one person. Minor RTIs are defined as no injuries at all to the involved individuals. Fatal RTIs are defined by death occurring at the crash scene or within one year and one day from injuries sustained from the crash. Limited 2009 data were available from the 2010 annual traffic report and were included if possible.

### 2.3. Inpatient Database

Casualty ward data from the emergency department at MNRH was collected over a one-month period (1–31 July) in 2015 and 2018. Information such as demographics (age and sex), date and reason of admission, type of trauma, district, tribe, and diagnosis were prospectively obtained by on-site research personnel who monitored patient progress.

### 2.4. Data Analysis

Data collection and analysis were performed with Microsoft Excel 2016. Descriptive statistics were calculated in the police traffic reports for the nature of RTIs, monthly/hourly trend of crashs, regional crash distribution, RTI causalities, age/gender distribution in RTIs, type of involved vehicle, RTI causes, and fined offenses. 

## 3. Results

### 3.1. Police Reports

#### 3.1.1. Demographics

**Figure 1:**
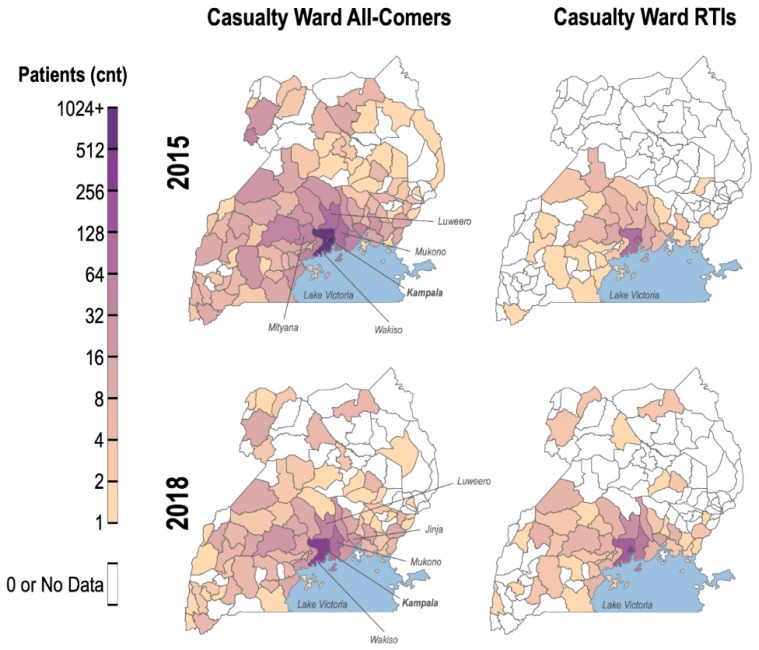
Heat map of RTIs by regions in Uganda.

Males were more frequently involved in RTIs than females (M: 73.95%, F: 26.05%). Averaged across all years and RTI severity, 74% of drivers involved in RTIs were male. The age group most involved in RTIs was 25–34 years old (Table 1). A large proportion of RTIs was localized to the national capital, Kampala (45.24%), and 60.71% in the central region, which encompasses Kampala (Figure 1). Of these RTIs, 22.90% were fatal, 48.86% serious, and 63.97% were minor. There was no significant difference in quantity or severity of RTIs between the wet (i.e., March–May, September–November) and dry (December–February, June–August) seasons (*p* = 0.93). Over one-third of fatal RTIs (37.49%) occurred between 6 p.m. and midnight, while serious and minor RTIs most commonly took place between noon and 6 p.m. (35.78%). The most common cause of RTI is careless driving, averaging 41.8% across all years. The second most common cause is reckless driving at an average of 30.3%. Causation trends are stable in spite of the gradual decrease in RTI across the years. Due to some categorization changes of causes across years, discrepancies can be observed in the less common causes (Table 2).

#### 3.1.2. Boda Boda Involvement in RTIs

**Figure 2:**
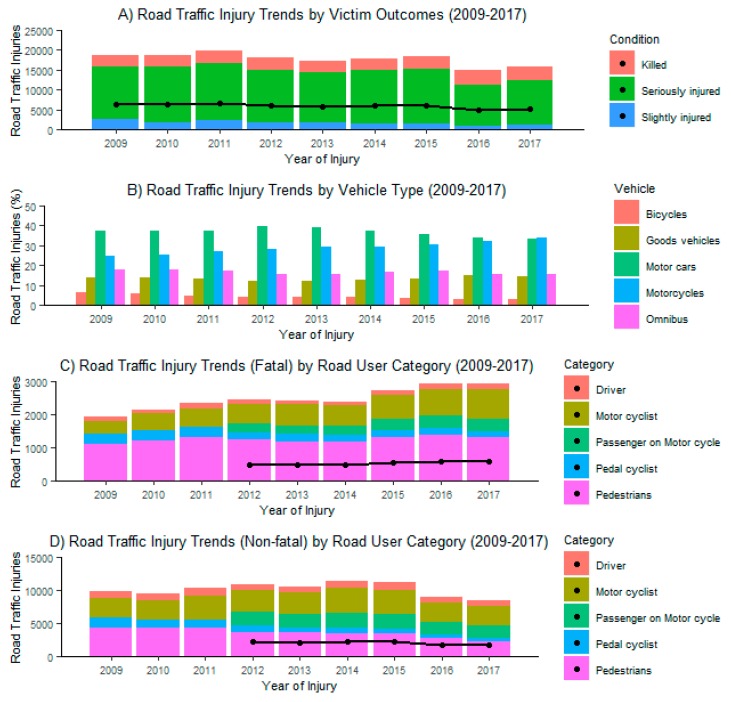
RTI trends from 2009 to 2017. Averages are overlaid as points. For C and D, “Passenger on Motorcycle” category was not available from 2009 to 2011, so averages were not calculated. (**A**): By victim condition: killed, seriously injuried, or slightly injuried (**B**): By vehicle type: bicycles, goods vehicle, motor car, motorcycle, or omnibus (**C**): Fatal injuries by road user: driver, motor cyclist, motorcycle passenger, pedal cyclist, or pedestrian (**D**): Non-fatal injuries by road user.

From 2009 to 2017, there was a steady increase in motorcycle (“boda boda”) RTI involvement from 24.5% to 33.9%. For comparison, the percentage of “motor car” involvement dropped from 37.6% to 33.6% in the same time period. Figure 2 shows trends for the top five classes of vehicles (motor cars, omnibus, goods vehicles, motorcycles, bicycles) involved in RTIs; only motorcycles have demonstrated consistent growth. By RTI severity category, motorcyclist involvement in fatal RTIs increased from 14.2% to 26.2%, in serious RTIs from 18.3% to 26.4%, and in minor RTIs from 15.6% to 19.3%.

#### 3.1.3. Mortality

The total number of annual road traffic crashes decreased from 22,461 in 2010 to 13,244 in 2017. The total number of victims decreased from 18,808 in 2010 to 15,752 in 2017, and the proportion of fatal RTIs increased from 14.7% to 22.2% (Figure 2). There was an acute 21.7% decrease in total RTIs between 2015 and 2016. During this time frame, the proportion of fatal RTIs doubled (11.6% to 22.8%), while the proportion of minor RTIs decreased from 44.4% to 27.7%.

### 3.2. Casualty Ward Database

During 1–31 July 2015, a total of 2962 patients (Female: 40%; average age of all patients: 35.4 years) presented to the casualty ward at MNRH (Table 3). Similarly, in July 2018, there were a total of 1306 patients (Female: 28%; average age of all patients: 30.1 years). In both cohorts, three-quarters of RTI patients (2015: 73.2%, 2018: 73.8%) reported a home address within 50 km of Kampala.

In 2015, RTIs (n = 299) accounted for 10% of all patients and 41% of all traumas. These percentages increased in July 2018, with RTI patients (n = 469) representing 36% of total patients and 64% of all traumas. Head traumas were documented in 54% of RTI patients in 2015 and 62% of those in 2018.

## 4. Discussion

The traffic landscape of Uganda has greatly changed within the past decade. In 2009, Uganda had 2989 kilometers of paved roads and 104,384 newly registered vehicles [23,24]. By 2017, paved roads increased to 4193 kilometers with 135,032 newly registered vehicles, reflecting 40.3% and 29.4% increases, respectively [23,24]. Given that per capita income (at 2009 constant price) has increased by almost 10% from 2011 to 2017, increasing economic growth has most likely translated to the increase in motor vehicle utilization and ownership. As Ugandan roads rapidly grow and fill with more drivers, there is even greater impetus to understand and address RTIs.

General observations of the nature of RTIs in Uganda can be made from our study. First, young males are most likely to be involved in RTIs, often taking place near the capital of Kampala during afternoon/night hours. Across the reported years, motorcycles are increasingly involved in RTIs, paralleling the increasing prevalence of Ugandan boda boda use and now even surpassing traditional motor cars. While RTIs have seen overall reductions in count, they have become more deadly, paralleling the increase in trauma patients due to RTIs in the casualty ward.

### 4.1. Trends in RTIs OverTtime

Our analysis provides a small window into the evolving nature of Uganda’s traffic safety and crash environment. The gross reduction in RTIs from 2010 to 2017 by 41.04% is encouraging. However, there has been a shift towards a higher percentage of fatal injuries, defined by the Ugandan police reports as death occurring anytime within one year and one day of the crash due to injuries sustained. Given these observations, a possible explanation is that motor vehicle usage increases have been matched with other factors (e.g., increased paved roads, familiarity, and experience with vehicles) that have increased safety and generally helped reduce overall RTI rates, minimizing the incidence of less severe injuries. However, when there are collisions, boda boda drivers are more likely to be involved in 2017 than in 2009. With greater risks already associated with motorcycles and low rates of helmet use, the higher rates of fatal injuries may be the consequence [5,7,8,25,26]. Interestingly, the distribution of RTI causes has remained consistent despite the RTI reduction (Table 2). This consistency is also observed with the young male demographic. Perhaps this could a specific target group for behavioral intervention counter careless and reckless driving [27,28]. Though our ability to interpret and draw conclusions from secondary data is limited, these trends call for greater scrutiny.

The MNRH casualty ward data in 2018 versus 2015 shows a greater proportion of patients presenting due to RTIs, both within the trauma cases (2018: 64%, 2015: 41%) and the overall patient population (2018: 36%, 2015: 10%). This may reflect a shift towards more severe injuries or increased access to emergency care. Additionally, an increasing proportion of RTI patients presented with head traumas, requiring neurosurgical evaluation. Given that there is already unmet need for surgery in Uganda, the advent of more trauma and neurosurgical cases risks further exacerbation of the surgical shortage [29].

The trends reflected in this study suggest a continued increase in RTIs requiring emergency, trauma surgery, and neurosurgery care. Addressing these traffic crashes thus requires a multi-faceted approach, including infrastructural improvements, traffic law enforcement, and investments into the healthcare domain to better address evolving trauma and neurosurgical requirements.

### 4.2. Literature Review of Ugandan RTI and Boda Bodas

The literature reiterates the burden of RTIs on Ugandan public health. At 28.9 deaths per 100,000, Uganda has a high RTI death rate compared to neighboring African countries. Additionally, RTIs rank among the top-ten causes of mortality in Uganda [30]. A cross-sectional retrospective study of MNRH in 2001 found boda bodas were responsible for 25% of RTIs [7]. A prospective study by Galkuande et al. of MNRH between 2004 and 2005 found boda bodas responsible for 28.6% of RTIs [5]. In 2011, a retrospective study of a Northern Uganda hospital found that injuries due to boda bodas were the second most common reason for admission due to trauma (21.8%) [8]. All studies also found that motorcyclists and victims were primarily young males [5,7,8]. Like the findings from the present study, these data confirm the growing role of boda bodas in crashes and confirm that observed trends have been persisting prior to the current study period. 

Though difficult to empirically assess, the authors present a variety of hypotheses regarding the propensity of boda boda involvement in RTIs. First, boda bodas are popular vehicles due to their cheap cost, especially to their young male audience [5,8]. With more boda bodas on the road, it is inevitable they will be connected to more RTIs. Second, poor enforcement of traffic regulations and laws leads to downstream effects on behavior. Many motorcyclists are ill-trained and have not undergone formal training, but many still drive lacking the proper permit without consequence [5,7,31,32]. Thus, there is no discouragement of over-speeding, reckless driving, and overloading with excessive passengers and cargo—all of which are common causes for RTIs. Additionally, as suggested by Kitara, many of these motorcyclists are self-employed as taxi-drivers, so there is a short-term economic incentive for aggressive driving to taxi more passengers [8]. Even safety measures are often unheeded, as evidenced by extremely low rates of required helmet use [25].

Contributory factors to motorcycle RTI occurrence and severity include the male sex, youth, inexperience, and lack of licensing [7,28]. These are all accurate characteristics of the current boda boda situation in Uganda and reflect its complex nature. Appreciating these circumstances is an important step to mitigate and prevent future RTIs.

### 4.3. Motorcycle Helmet Usage in Uganda

Boda bodas are the fastest-growing group of vehicles in Uganda [33]. Under Uganda’s Traffic and Road Safety (Motorcycles) Regulations, all motorcyclists and their passengers have been required, since 2004, to wear crash helmets while operating the vehicle [34]. However, as multiple studies have shown, adherence to helmet use is far from ideal [5,14,25]. This pattern of nonadherence is especially worrisome, given the increasing involvement of boda bodas in RTIs. 

By nature of the vehicle, motorcyclists and their passengers are far more exposed compared to car or bus operators. In police reports of RTI victims, motorcyclists and their passengers were more likely to have fatal and serious RTIs compared to car drivers (16.4% vs. 7.4%). Furthermore, the motorcyclists have a far higher percentage of injury compared to their passengers (20.2% vs. 12.7%).

The data from the MNRH casualty ward further elucidate the severity of these injuries: the percentage of RTI patients with head trauma reached 62% by 2018. It is possible that these data reflect a lack of helmet usage, exposing motorcyclists to greater risks of direct cranial injuries requiring emergent care. 

Alternatively, it is also possible that an increase in helmet usage led to more patients presenting to the hospital who otherwise would have died on impact. While both theories likely factor into increases of RTIs needing neurosurgical intervention, the reportedly low levels of helmet use suggest that the former explanation is far more contributory at this time [5,13,17].

Numerous attempts have been made to improve helmet usage by motorcyclists. Safeboda, an e-hailing company for boda bodas based in Kampala, enforces safety measures like helmets for its drivers. According to their reports, their drivers have a three-fold increase in proper helmet adoption (driver wearing a helmet and helmet being strapped), and their passengers have a 16-fold increase in adoption [35,36]. They found that incentives and proper training were effective ways to improve the adoption of safety measures. Safeboda’s findings suggest that private enterprise may be a powerful supplement to traditional governmental interventions. 

The Uganda Helmet Vaccine Initiative (UHVI), part of the larger Global Helmet Vaccine Initiative, has also sought to increase helmet usage in Kampala since 2010 [37]. It utilizes a combination of educational workshops, radio campaigns, free helmet donations, and establishment of helmet safety standards. From their observational studies, helmet use in Kampala increased from 31% in 2011 to 77% in 2015. These efforts were informed by important findings obtained from surveys of boda boda drivers’ attitudes towards helmets. From those interviewed, 74% knew of the requirement for helmets, and 71% had access to helmets [25]. The common complaints revolved around the helmet’s discomfort, poor quality, and expense. This data suggest that the onus of helmet usage may not be a lack of awareness, but rather the availability of adequate helmets.

### 4.4. The Impact of Government Road Safety Initiatives: Operation Fika Salama

Between 2015 and 2016, there was a noticeable drop in RTIs, according to police reports. In the “Nature of Crash” section, total RTAs dropped from 18,495 in 2015 to 14,474 in 2016, representing a 21.7% decrease; in comparison, percentage changes from the previous year 2013–2014 and 2015–2014 were only 1.7% and 1.0% decreases, respectively. Total victims dropped from 18,426 in 2015 to 14,854 in 2016, corresponding a 19.4% decrease; the changes from the previous year for 2013–2014 and 2015–2014 were only 3.3% and 3.2% decreases, respectively. This time period corresponds to a 2015 government road safety initiative called “Operation Fika salama” (FS). FS was implemented along major highways where police set up road blocks and fined people for driving violations. Although there is insufficient data to draw any correlation between FS and the drop in RTAs in 2016, the association is interesting and invites deeper analysis. Nevertheless, there is some circumstantial evidence regarding geography that may lend support to the possible correlation. According to the police reports, the top three regions corresponding to the highest numbers of RTAs are the eastern, southern, and northern portions of Kampala. Between 2015 and 2016, the combined RTAs from these regions had a 25.7% and 38.6% decrease for serious and minor injuries, respectively. In contrast, decreases in the previous year were only 6.5% for serious and 7.2% for minor injuries. Given that FS targeted major roadways, it is not unlikely that the Kampala RTAs from the reports were affected by the operation.

The 2017 and 2016 Ugandan police reports documented the various offenses that were fined during FS. Of the 20 possible offenses, only “Not wearing crush helmet” was reduced between 2016 (174 incidents) and 2017 (121 incidents). Again, there is insufficient data to judge if this decrease is significant, related to the efforts of Safeboda or UHVI, or simply an effect of enforcement. However, if interpreted optimistically, fewer offenses of not wearing helmets can indicate that efforts targeted towards boda boda drivers may be working.

### 4.5. Neurosurgical Care in Uganda

The increase of head trauma in RTI patients presenting at the MNRH casualty ward reinforces the importance of neurosurgical care in Uganda. Traumatic brain injuries (TBIs) are amongst the worst sequelae of RTIs and responsible for major morbidity and mortality [3]. And given Sub-Saharan Africa’s higher TBI incidence rate (compared to global rates), neurosurgery plays an acutely important role in minimizing its detrimental effects [3]. 

In Uganda, neurosurgical care has been improving, such as MNRH’s establishment of a neurosurgery intensive care unit in 2011 [9]. However, work is still ongoing to improve resources and care. Despite a non-inferior in-hospital mortality rate compared to high-income countries, MNRH experienced high mortality in patients whose surgeries were delayed [38]. Cost is another major issue—not only is the surgery potentially cost-prohibitive, but also the cost of a diagnostic CT scan may be equivalent to a month’s salary [29,38]. With our observations of increased boda-boda involvement in RTIs and the currently low compliance with helmet usage, more adequate resources for neurosurgery will be needed to care for TBI patients. 

### 4.6. Limitations

Our study has various limitations as an observational analysis. With the Ugandan police reports, our analysis is limited to what is included in the reports since we do not have access to the primary data. As mentioned previously, “killed”/“fatal” RTI cases are defined within a period of instant death up to one year and one day of the initial crash. Without access to the actual date of death, it is not possible to precisely correlate the reported “killed” patients with the casualty ward RTI patients. However, given the goal of reducing RTI morbidity and mortality, these data are sufficient in describing the context of Ugandan RTIs in broad strokes and underscore the importance of helmet usage for boda boda drivers. 

Additionally, MNRH’s casualty ward data does not account for all RTIs requiring medical care in Uganda. Despite having a broad catchment area, MNRH only captures a subset of RTI victims, while others may present at another hospital or not present at all. Furthermore, data collection was limited to the casualty ward and thus only provides a glimpse into the patient’s healthcare trajectory. Again, the goal of our study is not providing a perfect account of Ugandan RTIs, but rather the overall trends. The snapshot view of MNRH’s casualty ward is a complement to the analysis of the police reports during the same timeframe. In the future, a complete investigation in primary police traffic data and the casualty ward data may lead to more fruitful insights. While our descriptive study is unable to prove causality between observed relationships, it can serve as guideposts for more in-depth analyses in the future.

## 5. Conclusions

In the face of increased utilization of boda bodas’ in Uganda, helmet usage is becoming more important. In this study, we elucidate the evolving RTI trends in Uganda that demonstrate not only a gross reduction in RTIs but also increased motorcycle involvement in RTIs and an increased proportion of fatal injuries. These findings reveal a greater patient burden due to RTIs associated with a growing need for emergency, trauma, and neurosurgical care. The prevention of RTIs and reduction in their severity must be coupled with a strengthening of the care continuum necessary to address these injuries.

## Figures and Tables

**Table 1:** Annual Ugandan police traffic report demographics.

Category	2010	2011	2012	2013	2014	2015	2016	2017
Total casualties	18,808	19,955	18,016	17,283	17,848	18,435	15,754	14,854
Gender								
Male, n (%)	14,145 (75%)	14,785 (74%)	13,158 (73%)	12,709 (74%)	13,059 (73%)	13,558 (74%)	11,558 (73%)	10,862 (73%)
Female, n (%)	4663 (25%)	5170 (26%)	4858 (27%)	4574 (26%)	4789 (27%)	4877 (26%)	4196 (27%)	3992 (27%)
								
Age								
<18	2516	2634	2718	2388	2422	2480	2213	1934
18–24	16292 *	17321 *	3480	3078	3000	3298	2800	2553
25–34			5477	5640	6005	5808	5203	4747
35–44			3144	3083	3131	3274	2677	2435
45–54			1123	1111	1367	1391	1154	1173
55–64			392	412	467	599	484	574
65–74			198	190	206	252	225	328
>75			116	88	151	135	118	242
Unknown			1368	1293	1099	1198	880	868
								
Seasons								
Wet ^†^	11,207	10,870	9810	9384	9215	9272	6915	6725
Dry ^†^	11,254	11,402	10,060	8984	9471	9223	7559	6519
								
Time of RTI								
[0:00–5:59]	1811	2058	1992	1645	1585	1599	1309	1329
[6:00–11:59]	5872	5780	5096	4794	4815	4685	3630	3337
[12:00–17:59]	8280	8097	6782	6318	6511	6535	5037	4604
[17:59–23:59]	6498	6337	6000	5611	5774	5676	4498	3974
Wet season ^†^:	3–5, 9–11							
Dry season ^†^:	12–2, 6–8							
Age *:	adults (>18)							

*: 2010 and 2011 reports only designate adult (age >= 18) and non-adult (age < 18) categories. †: Wet season is months 3-5 and 9-11 | Dry season is months 12-2 and 6-8.

**Table 2:** Annual Ugandan police traffic report—nature of road traffic injury (RTI) for all vehicles.

Cause (%)	2009	2010	2011	2012	2013	2014	2015	2016	2017
Careless driving	9112 (42.0)	9316 (41.5)	8659 (38.9)	8694 (42.1)	7901 (41.0)	8708 (42.9)	8048 (40.6)	6567 (45.4)	5698 (41.6)
Careless pedestrian	889 (4.1)	881 (3.9)	1141 (5.1)	1079 (5.2)	1095 (5.7)	815 (4.0)	885 (4.5)	933 (6.5)	652 (4.7)
Dazzled by lights	85 (0.4)	114 (0.5)	152 (0.7)	46 (0.2)	25 (0.1)	28 (0.1)	44 (0.2)	49 (0.3)	150 (1.1)
Dangerous loading	-	-		82 (0.4)	93 (0.5)	94 (0.5)	117 (0.6)	56 (0.4)	162 (1.2)
DMC *	738 (3.4)	720 (3.2)	929 (4.2)	-	-	-	-	-	475 (3.5)
Obstacle on carriage way	96 (0.4)	76 (0.3)	266 (1.2)	59 (0.3)	3 (0.0)	4 (0.0)	6 (0.0)	1 (0.0)	118 (0.9)
Over loading	202 * (0.9)	240 * (1.1)	113 * (0.5)	29 (0.1)	78 (0.4)	85 (0.4)	58 (0.3)	30 (0.2)	161 (1.2)
Over speeding	973 (4.5)	839 (3.7)	867 (3.9)	308 (1.5)	492 (2.6)	677 (3.3)	954 (4.8)	588 (4.1)	461 (3.3)
Passenger falls	228 (1.1)	238 (1.1)	501 (2.2)	270 (1.3)	296 (1.5)	218 (1.1)	184 (0.9)	209 (1.4)	390 (2.8)
Reckless driving	6948 (32.0)	6579 (29.3)	6514 (29.3)	5831 (28.2)	5502 (28.6)	5670 (27.9)	5614 (28.3)	5140 (35.5)	4661 (33.9)
Under influence of alcohol	249 (1.2)	194 (0.9)	299 (1.3)	216 (1.1)	208 (1.1)	310 (1.5)	238 (1.2)	201 (1.4)	234 (1.7)
Unknown causes	2057 (9.5)	2129 (9.5)	1818 (8.2)	2971 (14.4)	2895 (15.1)	2985 (14.7)	2981 (15.0)	701 (4.8)	557 (4.1)
Weather/road conditions	112 (0.5)	1135 (5.0)	1013 (4.5)	1076 (5.2)	646 (3.4)	714 (3.6)	717 (3.6)	-	-
									
Total	21,689	22,461	22,272	20,661	19,234	20,308	19,846	14,475	13,719

DMC *: Dangerous mechanical condition; 2009, 2010, and 2011 do not differentiate by severity; Over loading and dangerous loading counted together.

**Table 3:** RTIs presenting at the Mulago National Referral Hospital (MNRH) casualty ward in July 2015 and July 2018.

Category	July 2015	July 2018	*p*-Value
Casualty ward population			
# of patients	2962	1306	
Gender			<0.001
*Male, n (%)*	1752 (59%)	937 (72%)	
*Female, n (%)*	1210 (41%)	369 (28%)	
Age, years	35.4	30.1	<0.001
Road Traffic Injuries			
# of patients	299	469	
% of casualty ward patients	10%	36%	
Age	29.4	29.6	0.817
Gender			0.235
*Male*	231	379	
*Female*	68	90	
Neurosurgery consult (n)	162	291	
% of RTI neurosurgery consult	54%	62%	

#: Total number.
